# Understanding and Managing Cognitive Distortions in Individuals With Schizophrenia

**DOI:** 10.7759/cureus.45268

**Published:** 2023-09-14

**Authors:** Kamal K Verma

**Affiliations:** 1 Psychiatry, K. D. (Kanti Devi) Medical College, Hospital & Research Center, Mathura, IND

**Keywords:** self-harm, violent behaviour, violent behavior, hallucinations, psychosis, psychotic symptoms, schizophrenia, delusions

## Abstract

Introduction: Schizophrenia is characterized by psychotic symptoms such as delusions, hallucinations, and disorganized thinking and speech. Patients suffering from schizophrenia incited by these delusions react violently in response to real or imagined threats; this engages them in violent behaviours and thus poses a threat. Sparse data are available for patients from India with regard to schizophrenia patients acting on their delusions. The aim of this study was to assess the prevalence of delusional action in patients suffering from schizophrenia and to identify the phenomenological characteristics of those delusions which are associated with action.

Material methods: This study was conducted on patients with a diagnosis of schizophrenia admitted to the indoor patient department (IPD) of the Department of Psychiatry, K.D. Medical College, Hospital & Research Centre, Mathura, India, during the period of February 2022 to July 2022. A semi-structured, semi-open-ended questionnaire was used for interviewing patients regarding demographics, the course of illness, past medical illness, the family history of psychiatric disturbances, and substance use. The study tool used for delusion was the Maudsley Assessment of Delusions Schedule (MADS).

Results: Out of 56 selected subjects, 34 acted on delusion and out of these, 19 were male and 15 female. In our study gender did not play any significant role in acting on delusion. Literacy and nuclear living households played a significant role in influencing delusion-driven behaviours, while the distinction between urban and rural living, though noteworthy, fell just short of achieving statistical significance. An emotional state like anger was significantly important to the patient’s acting on delusion, which led to violent behaviour or self-harm.

Conclusion: Positive responses are more likely to be associated with leading action on delusion as compared to negative responses, which were also associated with action on delusion; for example, anger was significantly important in the patient’s acting on delusion, which led to violent behaviour or self-harm.

## Introduction

Schizophrenia is a complex and chronic mental disorder that affects a person's thinking, perception, emotions, and behaviour, characterized by a combination of symptoms, including hallucinations, delusions, disorganized thinking and speech, social withdrawal, diminished emotional expression, and cognitive impairments [1]. Schizophrenia is estimated to affect about 0.3-0.7% of the global population. This means that approximately 20 million people worldwide have schizophrenia [2]. Its usual manifestation is in adolescents or in young adults, although it reaches its peak in the late teens to mid-30s for males and slightly later for females [3]. Although it affects both males and females, the age of onset is slightly early for males and may have more severe symptoms [3].

The genetic element plays a highly significant role in the development of schizophrenia. Higher risk is associated with people having first-degree relatives (parent, sibling, or child) with schizophrenia. Multiple genes are believed to play a role, in the development of schizophrenia. Various environmental factors can contribute to the risk of developing schizophrenia. These include prenatal and perinatal factors such as maternal infections during pregnancy, complications during childbirth, prenatal malnutrition, and exposure to certain toxins or substances. Increased risk of schizophrenia is also associated with growing up in urban areas, social adversity, migration, and exposure to childhood trauma or abuse [1].

Acting on these delusions poses a unique challenge for patients, caregivers, and healthcare professionals [4,5]. Acting on delusion refers to the behaviour exhibited by individuals when they succumb to false or irrational beliefs, often characteristic of conditions like schizophrenia or certain other mental health disorders. Delusions are persistent and firmly held false beliefs that are not grounded in reality, and acting on them can have a wide range of consequences, both for the individual and those around them. These actions can manifest in various ways, from harmless eccentricities to more concerning behaviours, such as self-harm or aggressive actions driven by paranoid delusions [6]. Understanding the factors that influence individuals to act on their delusions is crucial for mental health professionals and caregivers to provide appropriate support and interventions to help individuals manage their symptoms and lead fulfilling lives. Treatment strategies often involve a combination of medication, therapy, and support networks to assist individuals in reducing the impact of their delusions on their daily functioning [7]. The aim of this study was to assess the prevalence of delusional action in patients suffering from schizophrenia and to Identify the phenomenological characteristics of those delusions that are associated with action.

## Materials and methods

This is an observational cross-sectional of patients diagnosed with schizophrenia admitted to the indoor patient department (IPD) of the Department of Psychiatry, K.D. Medical College, Hospital & Research Centre, Mathura, India, during the period of February 2022 to July 2022. The selection was done based on the pre-decided inclusion and exclusion criteria. Patients aged between 18 to 65 years with at least one mood incongruent delusion were included in the study. Patients who refused to give consent, had severe formal thought disorders, and with co-morbid psychiatric illness and mental retardation were excluded from the study. The study was approved by the Institutional Ethics Committee of K.D. Medical College, Hospital & Research Centre (vide letter KDMCHRC/IEC/2022/01). Written informed consent was taken from all the participants.

A total of 72 subjects were examined. Of them, 56 were included in the study based on the selection criteria, including 30 males and 26 females. Diagnosis of schizophrenia was done based on the Diagnostic and Statistical Manual of Mental Disorders, Fifth Edition (DSM 5) [6]. The tool used for assessing delusions was the Maudsley Assessment of Delusions Schedule (MADS). The MADS, a semi-structured interview, evaluates the cognitive, affective, and behavioural aspects of delusions, along with self-reported evidence supporting these delusions. It categorizes the evidence for delusions into two categories: internal experiences (e.g., mood, anomalous experiences) and external events (e.g., actions of others). This assessment encompasses the phenomenology of abnormal beliefs such as conviction, preoccupation, and systematization, as well as the associated emotional responses. Additionally, it explores the subject's reasons for holding these beliefs, the resulting behaviours, and the subject's level of insight regarding the issue [7]. Moreover, the emotional state was measured using a questionnaire where individuals could rate their emotional experiences. The present study used the Positive and Negative Affect Schedule (PANAS) for the measurement of emotional experiences just before a subject acted on the delusion [5]. 

Based on the above tools, a semi-structured, semi-open-ended questionnaire was used for interviewing the patient regarding demographics, the course of illness, past medical illness, the family history of psychiatric disturbances, substance history and mental status examination. Statistical analysis was carried out using Python 3.10.0 software (Released 2021; Python Software Foundation, Wilmington, Delaware, United States). Statistical associations were tested using χ2 test. Significance was set at p<0.05.

## Results

Out of these 56, there were 32 males (57.14%) and 24 females (42.85%) (Table 1). A slight male predilection is observed in our data for schizophrenia. The average age of our data group was 38.6 years with females at an average age of 46.2 years and males at 35.43 years. Out of 56 participants, 34 acted on delusion (60.71%); out of these, 19 (33.92%) were males and 15 (26.78%) were females. The mean age of patients acting on delusions and those not acting on delusions was not statistically different (p-value=0.18). Also, no significant difference between married and unmarried subjects acting and non-acting on delusions was found in our study with p-values of 0.08, 0.20 and 0.94, 0.96, respectively. When the groups acting and non-acting on delusion were compared, no significant difference was noted in literate (able to read and write in any language) and illiterate participants with p-values of 0.67 and 0.51, respectively. When joint and nuclear families were compared, it was found that participants in the nuclear families were acting on delusions more as compared to those in joint families, with a p-value of 0.04. When we compared the ratio of acting and non-acting in males to females, no significant difference was noted with a p-value of 0.44 (Table 1).

**Table 1:** Demographic Variations and Acting on Delusion Data presented as Number (Percentage) unless marked otherwise

Variables		Acting on Delusion (n=34)	Non-Acting on Delusion (n=22)	P-Value
Gender	Male	19 (33.92)	13 (23.21)	0.28
	Female	15 (26.78)	9 (16.07)	0.22
Age (years), Mean ±SD		38.32±10.29	34.43±11.26	0.18
Marital status	Married	12 (35.29)	8 (36.36)	0.95
	Single	22 (64.70)	14 (63.63)	0.96
Education status	Literate	23 (67.64)	17 (77.27)	0.67
	Illiterate	11 (32.35)	5 (22.72)	0.51
Religion	Hindu	27 (79.41)	18 (81.81)	0.46
	Others	5 (14.70)	4 (18.18)	0.75
Family Type	Joint	11 (32.35)	7(31.81)	0.77
	Nuclear	23 (67.64)	15(68.18)	0.72
Domicile	Rural	12 (35.29)	9 (40.9)	0.74
	Urban	22 (64.70)	13 (59.09)	0.79

Table 2 compares the emotional status of both groups. It was found that emotional status does have an effect on acting on delusions. Subjects acting on delusion were significantly angry as compared to the ones not acting on delusion (p=0.00). Other emotions like elation, fright, depression, and anxiety do not have any significant difference statistically when both the groups were compared.

**Table 2:** Association of Emotion and Acting on Delusion Data presented as Number (Percentage); *p<0.05

Emotional state	Acting on Delusion (n=34)	Non-Acting on Delusion (n=22)	P-Value
Angry	15 (44.11)	1 (4.54)	0.00*
Elated/Happy	3 (8.82)	5 (22.72)	0.17
Frightened	18 (52.94)	11 (50)	0.88
Depressed/Unhappy	19 (55.88)	14 (63.63)	0.71
Anxious/Tense	16 (47.05)	12 (54.54)	0.69

Table 3 shows delusional actions reported by self, both positive and negative ones. It was found that 55.88% of the subjects who acted upon their delusions talked with someone about it as compared to 45.45% who did not act on their delusion, although the difference was not statistically significant. When an action like trying to prevent something was compared, it was found that it was significantly higher in the group that acted on delusion as compared to the non-acting group. Over 64% of the group that acted on delusion experienced anger, while the latter group had a lower incidence at approximately 40%. However, it's important to note that this difference did not reach statistical significance. It was observed that positive responses were more significant statistically in the first group (acting on delusion) with actions like feeling like hitting someone, actually hitting someone, or breaking something. Also, self-harm, which is an important action, was higher in the first group but this was not statistically significant; however, "tried to leave the house or move out due to X" was significantly higher in the first group (p=0.01). Among negative responses, no significant difference in both groups was observed. Hence, it is safe to say that positive responses are significantly higher as compared to negative responses when comparing the subjects on acting on delusion.

**Table 3:** Delusional Actions Reported by Self (Positive and Negative Responses) Data presented as Number (Percentage)

Actions–Positive response	Acting on Delusion (n=34)	Non-Acting on Delusion (n=22)	P-Value
Talked to anyone about X?	19 (55.88)	10 (45.45)	0.59
Tried to stop X happening?	7 (20.58)	0 (0)	0.03
X made lose temper	22(64.7)	9(40.9)	0.24
Feeling like hitting someone	13 (38.23)	2(9.09)	0.03
Actually, hit someone	6 (17.64)	0 (0)	0.04
Ever broken anything because of it	12 (35.29)	0 (0)	0.00
Tried to harm self or harmed accidently	3 (8.82)	0 (0)	0.16
Tried to move out or leave the house because of X	9 (26.47)	0 (0)	0.01
Have other changes resulted	1 (2.94)	0 (0)	0.42
Actions-Negative responses			
X stopped me from meeting friends	0 (0)	1 (4.5)	0.21
X stopped me from watching television	1 (2.94)	1 (4.5)	0.75
X stopped me from going to work	2 (5.88)	1 (4.5)	0.83
X stopped me from going to my doctor's clinic?	0 (0)	0(0)	-
X stopped me from doing things I would normally have done?	3 (8.82)	1 (4.5)	0.55

Table 4 is based on the questionnaire data received from the informant. It was found the altered dressing behaviour and damage caused inside or outside the house were the two actions that were significantly higher in patients who acted on delusions. Being violent toward someone was high but not statistically significant. Although harming self was less in number and significance, 8.82% of participants who acted on delusion showed this trait, which makes it alarming.

**Table 4:** Informant-Based Delusional Actions Data presented as Number (Percentage)

Questions	Acting on Delusion (n=34)	Non-Acting on Delusion (n=22)	P-Value
Has X been writing letters/emails or making telephone calls to unusual people?	3 (8.8)	1 (4.54)	0.55
Has anything he/she heard on television, radio or in the newspapers, during the past month, seemed to give rise to any odd or unusual behaviour or distress	7 (20.58)	4 (18.18)	0.84
Has there been any change in X’s eating and drinking habits? Has he/she been refusing food or drink?	22 (64.70)	12 (54.54)	0.63
Has X been behaving in the house in any other different or unusual ways?	11 (32.35)	8 (36.36)	0.80
Has X been feeling unsafe, frightened or scared at home and taking extra precautions, such as locking the door or putting a chain to the door?	22(64.70)	8 (36.36)	0.15
Has x been dressing in an unusual, inappropriate or different way?	10 (29.41)	1 (4.54)	0.04
Has X been suspicious of people recently? Has X been checking on anyone, or jealous of anyone	21 (61.76)	11 (50)	0.56
Has X damaged anything, either inside or outside the home?	8 (23.52)	0 (0)	0.02
Has X been violent to anyone	5 (14.70%)	0 (0)	0.07
Has X tried to harm him/herself	3 (8.82)	0 (0)	0.16
Has X been spending money in an extravagant or unusual way? If so, what on?	5 (14.70)	1 (4.54)	0.25
Has X been worried about his/her health? Has he/she visited the doctor or a hospital?	1 (2.94)	0 (0)	0.42

## Discussion

Delusional disorders and schizophrenia are forms of non-affective psychosis. Schizophrenia is characterized by hallucinations, negative symptoms, and cognitive symptoms more prominently as compared to delusional disorders. Acting on delusions in patients with schizophrenia is quite common (66.2%) [3]. Interestingly, self-reported actions were notably elevated in the case of individuals with schizophrenia. In a United Kingdom-based study, it was discovered that a significant 60% of participants exhibited such actions, a remarkably high figure [3]. Similarly, in our own study, 60.71% of the participants demonstrated behaviours stemming from delusions, a percentage closely mirroring the findings of Wessley et al. (60%) [8].

In our demographic analysis, we observed that the male-to-female ratio did not show statistical significance concerning the presence or absence of actions driven by delusions. Despite the higher prevalence of schizophrenia in males compared to females, our findings suggest that once individuals are diagnosed, gender does not appear to exert an influence on their behaviour [9]. Our results are in unison with Patel et al., who also did not find any significant difference in the acting of delusion of factors like marital status, age, gender, religion, or type of family (nuclear or joint). While comparing the groups, we found that the literacy and nuclear families had a role in acting on delusion with a significance of 0.0396 (p-value) but it was insignificant in the non-acting group [10]. Although the drawback of our study is the small sample size, further analysis with large data is required for this.

We found that more than 22.7% of non-acting subjects were more elated or happy with their beliefs as compared to a measly 8.8% of participants who acted out on their delusions. Also, anger was seen to be higher in the non-acting group as compared to the group that acted on delusion, which was the only significant trait of all the emotions. According to the research conducted by Patoilo on individuals with intermittent explosive disorder (IED), they similarly identified anger as one of the most frequently provoked characteristics [11]. Our results are also in comparison with the results of Patel et al. [10]. In our study, they were 60.1% whereas in studies by Patel et al. [10] and Kulhara et al. [12], it was 58.4% and 84.6%, respectively, which was in accordance with the other studies recorded in the literature [13-15].

Types of delusion associated with action specifically positive were significantly higher in our study, which was in accordance with Patel et al., although as per Buchanan et al., they did not reach a significant level and non-action was more as compared to action on certain beliefs [10,13]. In our study, we observed a notable increase in behaviours such as attempting to prevent events, experiencing impulses to strike someone, actual physical aggression, and instances of damaging objects. However, it's important to note that our findings diverged from those of the study by Buchanan et al. [13], particularly regarding negative actions, which showed no statistical significance in our research as well. No correlation was observed between the characteristics of delusional experiences and the act of acting upon those delusions when behavioural descriptions were provided by informants. However, when individuals described their own actions, acting on delusions was found to be linked with several factors, including awareness of evidence supporting the belief and active efforts to seek such evidence. Additionally, individuals who were more inclined to lessen their conviction when their beliefs were challenged, as well as those who experienced emotions such as sadness, fear, or anxiety as a result of their delusions, were more likely to engage in actions prompted by those delusions [13].

Of the participants who acted on delusions, 64.7% self-reported that delusions made them lose their temper while only 40.9% of participants who did not act on delusions self-reported it; based on reports by informants, 14.7% of the former group were violent on one or more occasions. Coid and Ullirich stated that most of the subjects who acted in violence were in a delusion of persecution, which made them angry, which is a warning sign of violent behaviour [16,17].

A total of 51.78% of subjects from our study talked to someone about their delusions; 55.88% of the subjects acting on delusion talked to some and 44.45% of non-acting participants talked to someone but the comparison of both groups was statistically insignificant. Our results tally with most of the studies found in the literature [1,8,9,12,18]. Safety-seeking behaviour like trying to protect oneself from the perceived threat was found significantly higher in the former group as compared to the latter. Of the participants who acted on delusions, 20.58% tried to prevent delusions from happening whereas none was seen doing so in the other group. This comparison was statistically significant with a p-value of 0.0333. Similar results were reported by Birchwood and Chadwick in their study [19]. The study provided validation for our cognitive model and therapeutic strategy. While the factors influencing the development of these core beliefs remain elusive, our discussion explores several hypotheses. One prevailing notion is that voice-related beliefs may evolve as an adaptive response to the presence of auditory hallucinations, rooted in fundamental beliefs about self-worth and interpersonal schema [19].

Of subjects who acted on delusions, 26.4% tried to leave the house or move out but none reported the same in the other group; this was again statistically significant with a p-value of 0.015. These results are in unison with the systematic review of safety-seeking behaviour and acting on delusions conducted by Tully et al. [20]. In relation to the persecutory beliefs, 100% of subjects reported using self-safety behaviour (SSB) in the studies conducted by Garety et al. [21], but in our study, only 47.05% of the subjects acting on delusions adopted SSB, and our results are in accordance with Patel et al., Kulhara et al., and Jakhar et al. [10,12,22]. None of the participants who did not act on delusions resorted to SSB, which was in unison with the results of Patel et al. [10]. The above differences in the studies could be due to the selection of subjects. For example, in our study, we selected diagnosed cases of schizophrenia but in Freeman et al.'s study, cases of psychosis were selected [23]. Also, the definition of SSB is quite broad and it is possible that the MADS does not include all the questions of the Safety Behaviours Questionnaire [23]. Garety et al. set out the cognitive processes that lead to the formation and maintenance of the positive symptoms of psychosis and they attempted to integrate into a model research in social factors [21].

Within the group that acted on delusions, a mere 8.82% of participants reported instances of self-harm, and these individuals exhibited some degree of insight into their actions. Conversely, self-harm was absent in the other group, although statistical significance was not achieved, possibly owing to the limited sample size. Notably, a separate study from India reported a higher self-harm risk of 22.6% [22]. This variation could be attributed to disparities in parameters and the choice of assessment scales. Suicide and violent behaviour are statistically significant with acting on delusions in our study; this is in accordance with Freemen et al. [23]. Our study aligns with findings from Cheung et al., where an escalation in violent behaviour was linked to elevated anxiety levels [24]. Cheung et al. noted that this increase in violence was primarily a result of negative emotional reactions, including anger, anxiety, or sadness. These emotional responses had a more pronounced impact on violent actions compared to the intensity of symptoms, and our study corroborates these findings.

We did not find the level of insight to predict acting on delusions, which differs from the study conducted by Patel et al. and Buchanan et al. [10,13]. Of our study subjects, 85.29% were frightened but there was no significant difference in the groups on the comparison. Our results tally with Patel et al. but do not agree with Buchanan et al. [10,13].

Limitation of study

The study's limitations include a relatively small sample size and its exclusive location in an urban setting, which predominantly featured literate individuals from middle-class backgrounds, potentially biasing the results. Additionally, the cross-sectional nature of the study inherently constrains its overall potential for drawing comprehensive conclusions.

## Conclusions

Acting on delusions does not have any gender bias. Emotional states such as fear, anxiety, and depression are more likely to result in acting on delusion. Literacy and nuclear living have a significant role in leading to actions on delusion. We also conclude that positive responses are more likely to be associated with leading action on delusion as compared to negative responses and the delusion of persecution was the most common delusion in our study, which was also associated with action on delusion.
